# The synergistic action of imidacloprid and flumethrin and their release kinetics from collars applied for ectoparasite control in dogs and cats

**DOI:** 10.1186/1756-3305-5-73

**Published:** 2012-04-12

**Authors:** Dorothee Stanneck, Ulrich Ebbinghaus-Kintscher, Eva Schoenhense, Eva M Kruedewagen, Andreas Turberg, Andrew Leisewitz, Wolfgang Jiritschka, Klemens J Krieger

**Affiliations:** 1 Bayer Animal Health GmbH, D-51368 Leverkusen, Germany; 2 Bayer CropScience AG, D-51368 Leverkusen, Germany; 3 Department of Companion Animal Clinical Studies, Faculty of Veterinary Science, University of Pretoria, Department of Companion Animal Clinical Studies, Private Bag X04, Onderstepoort, Pretoria 0110, South Africa

**Keywords:** Imidacloprid, Flumethrin, Collar, Cat, Dog, Synergism, Efficacy, Slow release

## Abstract

**Background:**

The control of tick and flea burdens in dogs and cats has become essential to the control of important and emerging vector borne diseases, some of which are zoonoses. Flea worry and flea bite hypersensitivity are additionally a significant disease entity in dogs and cats. Owner compliance in maintaining the pressure of control measures has been shown to be poor. For these reasons efforts are continuously being made to develop ectoparasiticides and application methods that are safe, effective and easy to apply for pet owners. A new polymer matrix collar has recently been developed which is registered for 8 months use in cats and dogs. The basic properties of this collar have been investigated in several *in vitro *and *in vivo *studies.

**Methods:**

The effects of imidacloprid, flumethrin and the combination were evaluated in vitro by means of whole cell voltage clamp measurement experiments conducted on isolated neuron cells from *Spodoptera frugiperda*. The in vitro efficacy of the two compounds and the combination against three species of ticks and their life stages and fleas were evaluated in a dry surface glass vial assay. The kinetics of the compounds over time in the collar were evaluated by the change in mass of the collar and measurement of the surface concentrations and concentrations of the actives in the collar matrix by HPLC. Hair clipped from collar treated dogs and cats, collected at various time points, was used to assess the acaricidal efficacy of the actives ex vivo.

**Results:**

An *in vitro *isolated insect nerve model demonstrated the synergistic neurotoxic effects of the pyrethroid flumethrin and the neonicotinoid imidacloprid. An *in vitro *glass vial efficacy and mortality study against various life stages of the ticks *Ixodes ricinus, Rhipicephalus sanguineus *and *Dermacentor reticulatus *and against the flea (*Ctenocephalides felis*) demonstrated that the combination of these products was highly effective against these parasites. The release kinetics of these actives from a neck collar (compounded with 10% imidacloprid and 4.5% flumethrin) was extensively studied in dogs and cats under laboratory and field conditions. Acaricidal concentrations of the actives were found to be consistently released from the collar matrix for 8 months. None of the collar studies in dogs or cats were associated with any significant collar related adverse event.

**Conclusion:**

Here we demonstrated the synergism between the pyrethroid flumethrin and the neonicotinoid imidacloprid, both provided in therapeutically relevant doses by a slow release collar matrix system over 8 months. This collar is therefore a convenient and safe tool for a long-term protection against ectoparasites.

## Background

The control of ectoparasites is crucial in the prevention of various vector-borne diseases that cause high morbidity and in some cases mortality in dogs and cats. Some of these infections are zoonoses and hence ectoparasite control on dogs and cats has become a major concern [1-5]. Ticks are second only to the mosquito as a means of disease transmission in humans and the most important vector of vector-borne disease in dogs [6]. Pet travel is now commonplace and as such many vector borne diseases cross borders causing diseases atypical of their traditional geographic distribution with the result that many dog tick borne diseases are now recognised as emerging disease threats [1]. Over the last few decades several new tick-borne infections have been identified and others are regarded as re-emerging diseases (especially in dogs) [1]. Acaricides and insecticides have typically been applied as dips and rinses, shampoos, powders, systemically administered tablets, spot ons, sprays and chemically impregnated collars [7].

The ideal ectoparasiticide product would be one with a broad acaricidal and insecticidal activity, would contain an active ingredient or combination of ingredients that are non-toxic to pets, humans and the environment, would be easy and simple to administer and would have a very long residual action. Break down in ectoparasite control strategies occur frequently and one of the most common reasons for this is poor owner compliance causing disruption and sporadic use of control measures. There is some work to show just how poor owner compliance actually is in the care of the veterinary patient[8-12]. Interestingly enough, there is only one study that assesses owner compliance with regard to the use of ectoparasiticides [13]. In this study where 1271 dogs were evaluated for tick and flea preventative data, approximately 74% of dogs were being treated with a tick and flea control product and only 61% used these products year round. 138 cats were evaluated for the use of tick and flea preventative. Only 38% of this cat population were using tick and flea preventatives, with 47% using a product year round and 34% using a product seasonally. The use of products was varied according to season with owners being more diligent during what they perceived as being the high risk seasons [13]. In some countries (where there are distinct seasons) disease transmission is seasonal but in the areas of the world with more temperate climates or in the subtropical or tropical regions, disease transmission is a year round problem. It has been shown that the simpler a treatment protocol is, the more likely an owner is to comply [11]. Poor owner compliance has also been seen as possibly due to veterinarians relying increasingly on broad spectrum anti-parasitic drugs and very short consultations. This results in poor client education which is an important factor resulting in poor client compliance [14]. This trend is all the more reason why products used should be easy to apply and robust in efficacy in the hands of pet owners who are unlikely to be given consistent and clear guidance from a veterinarian in regards to ectoparasite control [13,14]. Acaricide and insecticide impregnated collars have been widely used in an attempt to simplify the administration of ectoparasiticides in dogs and cats [15-18].

The pyrethroid flumethrin has been registered for animal use as an acaricide since 1986. Its pharmacological activity is mediated through voltage gated sodium channels in neural tissue which it causes to remain open for longer than physiologically normal, thus extending the period of sodium influx [19]. The effect will only be seen in stimulated tissue; resting tissue is unaffected by the compound. Once stimulation occurs however, the result is repeated shorter or longer bursts of nerve impulse resulting in death of the organism [20]. It has minimal effect on mammals as it shows a high specificity for invertebrate neural tissue. It has been extensively used in livestock as an acaricide but is also registered for use in a collar preparation in combination with propoxur [18]. Imidacloprid, discovered in 1984, is a highly effective insecticide and has become the largest selling insecticide worldwide for agricultural and veterinary use in the last decade [21]. It has its pharmacological action at the insect nicotinic acetylcholine receptor (nAChR) on the post synaptic membrane where it interferes with neurotransmission by causing prolonged opening of the sodium channel resulting in sustained depolarization of the neuron and death [21]. It is highly insect specific in this action and has minimal effect on the mammalian nAChR [22,23]. Imidacloprid has been registered for use in dogs and cats as a spot on product [21]. It is also available in combination with permethrin (also a pyrethroid) for spot on use in dogs [24].

This report demonstrates the use of a unique active ingredient combination of imidacloprid (10%) and flumethrin (4.5%) which show significant synergism in an *in vitro *isolated insect nerve study and in a glass vial efficacy and mortality study with various tick species and fleas. The formulation of imidacloprid and flumethrin in a collar matrix proved safe to dogs and cats and resulted in a very prolonged release of both compounds which indicates that pet owner compliance could be made significantly more simple with a single collar application lasting as long as 8 months.

## Methods

### Care of experimental animals

Laboratory based studies which were the source of the collars used for the release kinetics investigations were conducted at two sites (Bayer Animal Health, Leverkusen, Germany) and ClinVet International (Pty) Ltd., Bloemfontein, South Africa. All studies were done in compliance with Good Clinical Practice (GCP) and/or Good Laboratory Practice (GLP) and in compliance with the standards and norms of international legislation in regards to animal use and care. Animals were either identified by permanent tattoo or subcutaneously placed microchip transponders. All animals were adequately acclimatised before entering a study. All animals were fed commercial rations and had their health status checked by a veterinarian before and during all trials. The efficacy results of these studies are reported by Stanneck [25,26].

The field studies were conducted as multicentre dog and cat studies in multiple veterinary clinics in Germany, France, Hungary and Portugal. The field studies were conducted in compliance with Good Clinical Practice and in compliance with the standards and norms of international and national legislation for field studies. Animals were living in their normal household environments as pets and had been presented to the study veterinarians for health care. Their participation was voluntary. All animals had their health status checked by a veterinarian before and during the studies. The efficacy and safety results of these studies are reported by Stanneck [27].

### Synergism between flumethrin and imidacloprid

#### Whole cell voltage clamp measurements on isolated neuron cells from spodoptera frugiperda

Whole cell voltage-clamp electrophysiological recordings were performed on isolated neuronal cell bodies obtained from *Spodoptera frugiperda *larval ganglia after enzymatic treatment and mechanical dissociation. The ganglia were treated with 0.002 g/L dispase (GIBCO BRL 17105-041), incubated for 5 min at 37°C, centrifuged, and resuspended in culture buffer (based on supplemented Leibowitz L-15 medium (Invitrogen 11415064) (for details see [28]) by gentle aspiration with a fire-polished pasteur pipette (Hilgenberg, Malsfeld Germany), with slight modifications as described elsewhere [29]. Cell somata were plated onto glass cover slips previously coated with concanavalin-A (400 μg/ml, Sigma C2010) and laminin (4 μg/ml, Sigma L2020). The cells were kept at room temperature. Electrophysiological recordings were done with the whole-cell voltage and current clamp technique as described elsewhere [30]. The external bath contained Ringer's solution (in mM): 150 NaCl, 4 KCl, 2 MgCl2, 2 CaCl2, 10 HEPES (pH 7.4 adjusted with NaOH). The (internal) pipette solution contained (in mM): 120 CsF, 30 CsCl, 10 Cs-EGTA, 1 CaCl2, 10 HEPES (pH 7.4 adjusted with CsOH). Compounds were applied to the cells using the U-tube reversed flow technique [31]. Imidacloprid and flumethrin were synthesised in house. The test compounds were freshly dissolved as a 10 mM stock solution in DMSO (Sigma D8418) and diluted to the required concentrations in Ringer's solution before the experiment. Currents were measured with an L/M-EPC 7 patch clamp amplifier (List, Darmstadt, Germany). Current records were low-pass Bessel filtered at 1 kHz and digitized at 3 kHz sample rate for measurements on voltage gated Na^+ ^channels.

#### Extracellular recording of spike activities on isolated nerve cords from spodoptera frugiperda

Abdominal ganglia with a length of the associated distal nerve cord were prepared from *Spodoptera frugiperda *larvae and placed into a two-part plexi-glass chamber filled with Ringer Solution (in mM: 150 NaCl, 4 KCl, 2 CaCl2, 2 MgCl2, 10 HEPES, pH 7,4). The gap between the chambers was sealed with petroleum gel (Vaseline^®^). The caterpillar ganglia prepared in this way could be used for two hours for electrophysiological experiments. To monitor spike activity in the nerve cord, the voltage difference between the two chambers was measured with a differential amplifier (DAM-50, World Precision Instruments, Berlin, Germany). Whenever an action potential propagated along one of the axons in the nerve cord under the petroleum gel barrier, a transient biphasic potential of a few tens of microvolts could be measured between the two chambers. These signals were viewed on an oscilloscope and recorded through a digitizing interface (PowerLab, AD Instruments, Spechbach, Germany) into a PC running the PowerLab Chart Recording software. Sample recordings were taken as screenshots from the computer monitor and pasted into the figures. Spike counts were obtained with the frequency counter feature of the Chart Recording Software. A threshold was set arbitrarily to recognize distinct spike activity in the first baseline recording, and the counter was then viewed during the replay of the recordings for the entire experiment. In the recording chamber, the ganglion was bathed in Ringer Solution. Drug solutions were applied by rapidly exchanging the solution in the chamber (volume: 800 μl) and allowing the drug to diffuse into the ganglion for 3-5 minutes. Washout was done by exchanging the chamber volume twice in succession with Ringer's solution. Flumethrin and imidacloprid were synthesized in house. Flumethrin was dissolved as a 10 mM stock solution in DMSO (Sigma, D8418) and freshly diluted in 1:10 in DMSO to enable the required test concentrations in Ringer's solution with a final DMSO concentration of 1% before an experiment. Imidacloprid solutions were prepared from the 10 mM DMSO stock solution and diluted to the required test concentrations in Ringer's solution.

### *In vitro *evaluation of the efficacy of imidacloprid - flumethrin combination against ticks and fleas

#### Imidacloprid flumethrin coated glass vial preparation

Glass vials for both tick and flea studies were prepared as follows: Compound was dissolved in acetone p.A. (Merck KGaA, no. 1.00014.1000, 99.8%) and diluted in the same solvent. 3 × 250 μl of each compound dilution was transferred into three rolled edge snap cap glass vials vials (25 ml snap-on lid flask ND22, 65 × 26 mm, Fisherbrand, art. no. 320 55 60) with an acetone-primed pipette. Vials were placed on a Test-Tube Rotator (Rock 'n' Roller, model L-202, Labinco BV, NL) at 10 rpm and 5 rocking cycles per minute for at least 2 hours at room temperature under a fume hood. Acetone was allowed to dry off leaving a homogeneous coating of active on the glass vial walls and bottom. The total surface area of the glass vial was 44.7 cm^2^. With 250 μl from a 900 ppm solution approximately 5 μg of active were applied per cm^2^. Where the two ingredients were used in combination, the dose rate of the more highly concentrated active was 900 ppm. The second ingredient was added at a lower dose according to the mix ratio required (here 1.85 parts imidacloprid: 1 part flumethrin). Thus, at 900 ppm imidacloprid, flumethrin was present at 486 ppm. The ratio of 1.85:1 imidacloprid:flumethrin was chosen as this was what was found to be the ratio of these two compounds in animals hair coat during hair coat kinetic studies performed using these compounds in a slow release collar device (see later).

#### Coated glass vial contact assay against ctenocephalides felis

Adult male and female cat fleas (*Ctenocephalides felis*) from a laboratory colony susceptible to neonicotinoids and synthetic pyrethroids (adapted to breeding in an *in-vitro *feeding system but also reared on colony cats) and not more than 5 days post emergence were used. Fleas were kept at room temperature and 80% relative humidity until sorting and use in the experiments.

A portion of the fleas were anesthetized for 3-5 seconds with CO_2 _and approximately 10 adult male and female fleas of random gender ratio were transferred to each vial as soon as they were fully immobilized. Vials were closed with an untreated snap cap with needle-punctured holes in the centre. Vials were kept at ambient temperature and humidity horizontally to ensure maximum contact between fleas and glass vial surface.

All original test data (represented as means from two experiments) were analysed for statistical difference by means of a paired two-tailed *t*-test after being tested for normality. Significance was set at *p *< 0.05.

After 24 and after 48 hours flea activity was monitored after gently tapping the vials on a hard surface. Vials where all fleas showed normal occasional jumps and coordinated movements were evaluated as zero efficacy. Fleas with uncoordinated movements or fleas that lay on one side showing only weak leg movements were counted as alive despite the fact that they would not be capable of infesting or feeding on a host. Fleas not moving at all after stimulation by tapping the vial were counted as dead. Untreated and solvent treated control vials served as controls.

#### Coated glass vial contact assay against various life stages of ticks ixodes ricinus, rhipicephalus sanguineus and dermacentor reticulatus

Assays against all three life stages (larvae, nymphs and adults) were conducted for *I. ricinus *and *R. sanguineus *whilst *D. reticulatus *larvae and adults were evaluated in this glass vial contact study. In all cases laboratory colonies were used at least 14 days post emergence. All ticks were kept at room temperature and 90% relative humidity until sorting and use. *Ixodes ricinus *and *Dermacentor reticulatus *developmental stages were also kept at 90% relative humidity throughout the incubation period of the coated vials.

Five active adult ticks, 10 nymphs (no nymphs were available from *D. reticulatus*) or approximately 25 larvae were selected and transferred to each glass vial (vials were prepared as described previously). Vials were closed with an untreated snap cap with needle punctured holes in the centre. Vials were stored horizontally and in the dark to avoid movement of ticks onto the untreated lid.

After 24 and 48 hours ticks were forced to the bottom of the glass vials by gently tapping the vials on a hard surface. Unlike fleas, ticks tend not to move voluntarily and hence a temperature stimulus at a temperature triggering typical tick heat avoidance behaviour was used to evaluate their viability: Glass vials were transferred onto a heating table (at 45-50°C) for a maximum of 15 minutes. No more than 3 vials were handled at any one time. Vials where all ticks showed normal heat avoidance and coordinated movement were evaluated as having zero efficacy. Ticks with slow heat avoidance and uncoordinated movements or absent heat avoidance and poor leg and/or mouthpart movement were counted as knock-down ticks (judged incapable of normal host seeking and feeding). Ticks not moving at all and not stimulated by an additional carbon dioxide pulse were regarded as dead. Only mortality counts were used in this study. Ticks from untreated and solvent treated control vials served as controls. All original mortality test data were analysed for statistical difference by means of a paired two-tailed *t*-test after being tested for normality. The Wilcoxon Signed Rank Test was used if the probability of normality was below 0.05. Significance for both tests was set at *p *< 0.05.

### Release of active ingredients over time

#### Weight loss of collars over time

The release of active ingredients from the collar as evaluated by collar weight change was studied in both cats and dogs. Two collar sizes were used. A small collar (38 cm long) used in cats, and in dogs weighing 8 kg or less. A large collar (70 cm) was used in dogs weighing more than 8 kg. A total of 177 dogs were collared under laboratory conditions across 11 different studies and 59 dogs were collard under field conditions in a single study (thus a total of 236 collars were evaluated). The cat studies involved 9 laboratory based studies including 157 animals and a single field study involving 66 animals (thus totalling 223 cats). Collars were evaluated over a period of 240 days. Both dog and cat collars contained the same amount of active ingredients (imidacloprid 10% and flumethrin 4.5%; on a per collar weight basis 1 g of collar contained 101 mg imidacloprid and 45 mg flumethrin). Collars were weighed once they had been trimmed to size and the application weight was used as the base line weight against which all changes in weight were measured. In this way the change in collar weight could be calculated based on weight loss over time.

#### Surface content of actives on the collar over time

The surface content of actives on collars compounded with 10% imidacloprid and 4.5% flumethrin was evaluated at various time points after they had been worn by cats. All cats were European short hair breed that entered the trial at 2 years and 32 months of age. Weights varied between 3.3 and 5.1 kg on entry to the study. Three laboratory housed cats per group (6 groups in total) were fitted with a collar and after a period of 2, 7, 14, 28, 56 and 84 days the collars were removed for analysis. All collars were weighed before application and upon removal. The detection and quantification of the active ingredients imidacloprid and flumethrin on the surface of the collars was performed as follows: a piece of collar was placed in a suitable flask and agitated in a solvent mixture (acetonitrile and water (8:2) for a defined time on a rotary shaker. Actives were detected and quantified by HPLC using gradient elution (acetonitrile and water with 0.01%TFA) on a 150*4.6 mm stainless steel column, packed with 4 μm RP18 material. For detection a UV-spectrophotometer at 268 nm was used. Quantification was calculated against an external standard. The surface release was calculated in mg/g of collar.

#### Release of active ingredients over time

Weight loss of the collars is not a true reflection of the release of the active ingredients alone but also will be partially due to loss inert components such as plasticizers. For determination of the active ingredient released over time, 177 randomly chosen collars that had been worn for different time periods (up to 8 months) by cats and dogs in various efficacy and target animal safety studies as well as in field studies were analysed to determine their remaining active ingredient content. The determination of active ingredients in the collar body was performed by HPLC after complete dissolution of the collar in tetrahydrofuran (THF) and removal of plastics via precipitation by acetonitrile. HPLC was then performed as described above. The amount of active ingredient remaining in the collar was calculated against the nominal start content of both active ingredients in the collar.

### Acaricidal efficacy of cat and dog hair over time

The acaricidal activity of the actives on hair harvested from dogs and cats in long term hair coat kinetics studies was investigated (Bayer Animal health study ID 35642 and ID 35643, unpublished data). Hair was harvested from 8 dogs and 8 cats that were wearing 10% imidacloprid/4.5% flumethrin collars. Hair samples were collected with scissors (cutting as close to the skin as possible) from the animals' lateral left and right thoracic region on days 7, 14, 21, 30, 59, 90, 120, 149, 181, 210 and 240. From each of these eleven time points, a representative hair sample was selected which had a flumethrin content nearest to the group mean at the time point. This reduced inter-individual variability. 0.1 gram of each sample (left and right side) was weighed out, mixed together and placed in individually marked Petri dishes (diameter 6 cm). Samples of the same animals were taken at two pre-treatment time points and served as negative untreated controls. A total of 6 adult unfed *Rhipicephalus sanguineus *ticks (3 male and 3 female) originating from the Bayer colony were counted into glass vials three days before the test and kept in an incubator at a temperature of approximately 25°C and a relative humidity of 70-80% until the test day. On the test day the content of one vial was placed into each Petri dish. The Petri dishes were incubated under the same conditions until examination. After a contact time of 4 h, 6 h, 12 h and 24 h the number of dead ticks, moribund ticks, ticks with uncoordinated movements and live ticks were counted. If the differentiation was not possible with the naked eye, a stereomicroscope and a heating plate (temperature 40 ± 5°C) was used for detailed examination. Efficacy was determined by comparison of the live tick counts after each of the different contact times and compared to the control samples taken before treatment. The percent efficacy was calculated according to the recommendations for controlled tests described in the guideline EMEA/CVMP/005/00-Final [32]

$$\%\text{ Efficacy }=\;\left(\text{N2}-\text{N1}\right)/\text{N2 }\times \text{ 1}00$$

N1 = number of ticks(treatment failure) in the group treated with IVP

N2 = Number of ticks (treatment failure) in the pre-treatment samples

Two versions of efficacy were calculated:

1) *Killing efficacy *was calculated using dead or moribund ticks (treatment was regarded as a failure if all ticks were found alive or in a moribund state).

2) *Overall efficacy *was calculated using all dead, moribund and uncoordinated/ataxic ticks (treatment was regarded as a failure if all ticks were found alive).

## Results

### Mode of action of pyrethroids on the insect Na^+ ^channel. Demonstration of the"use dependent" effect of pyrethroids

**Figure 1:**
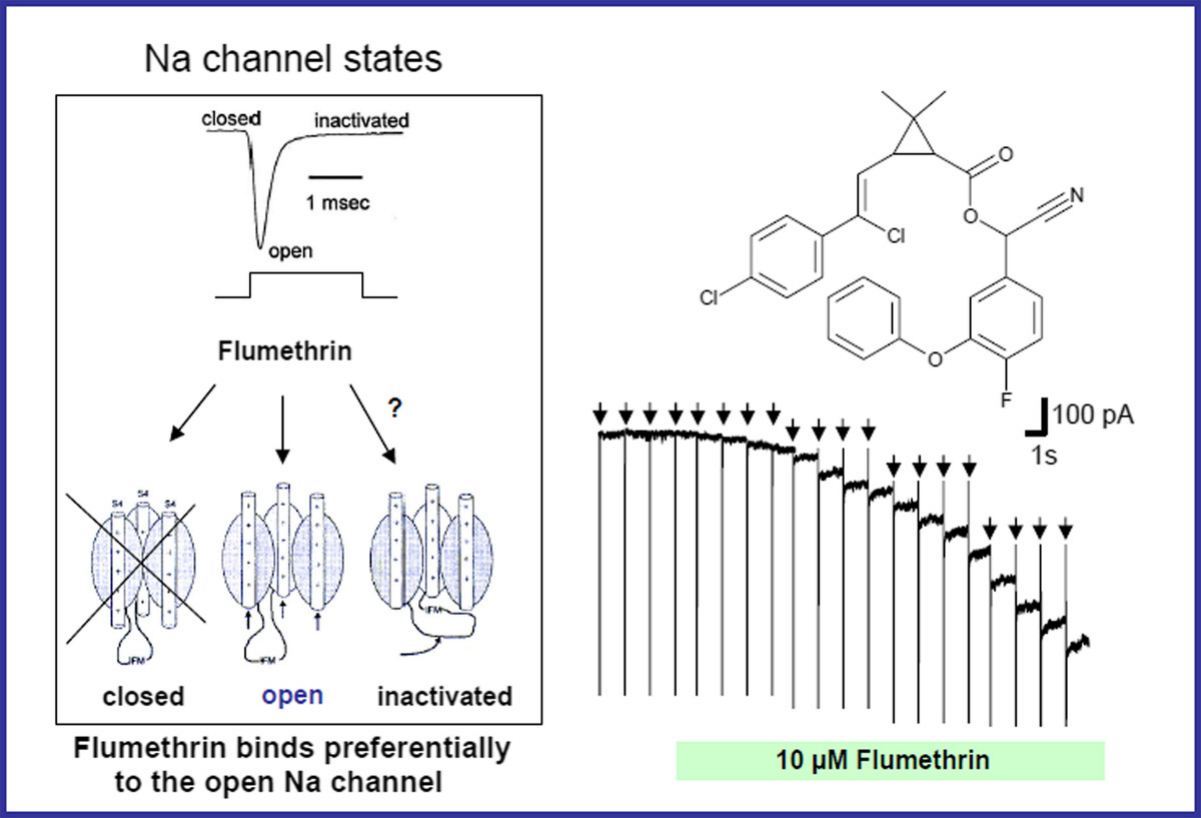
**The use dependent effect of flumethrin**. Figure 1a illustrates the three states of the voltage-gated sodium channel (closed, open and inactivated). Flumethrin binds preferentially to the open state of the Na-channel and keeps the channel open as indicated by the experiment shown in Figure 1b: In this experiment, flumthrin is applied (10 μM, green bar) to an isolated insect nerve cell in the whole cell voltage clamp configuration (holding potential Vc = -100 mV, Na^+ ^channels closed). During the experiment 20 short current pulses (downward arrows, every 1250 ms) were applied to change the holding potential from -100 mV to -10 mV to activate the voltage-gated Na channels (the thin downward lines reflect the Na currents induced by opening of the Na channels). Because flumethrin caused a downward change in the membrane current after Na^+ ^channel opening only and not during the much longer resting phase, this experiment clearly demonstrated the use-dependent effect of flumethrin. (Figure 1a modified from: reference [33]).

On the insect nerve cell preparation from *Spodoptera*, flumethrin bound preferentially to the open (activated) state of the Na^+^-channel and acted to keep the channel open as shown in Figure 1a and 1b. Application of 10 μM flumethrin had almost no effect on the resting nerve cell when the Na^+ ^channels were closed (as represented by the space between the arrows). Only after channel opening, induced by a single short current pulse (mean open time < 2 ms, each downward arrow representing a pulse) could flumethrin bind to the channel and prevent channel closing (i.e. the channels modified by flumethrin stayed open), which caused an increase of the membrane current. This demonstrated the "use dependent" effect of pyrethroids.

### Mode of action of imidacloprid on insect nAChR

**Figure 2:**
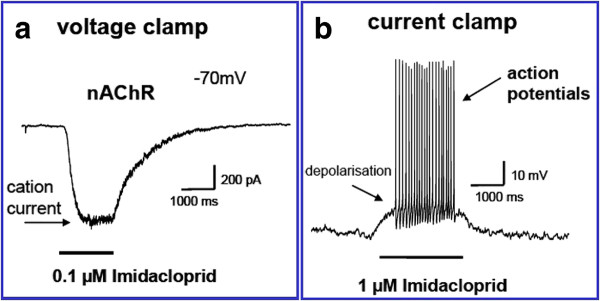
**The effect of imidacloprid on an isolated nerve cell in the whole cell voltage clamp mode (a) and the current clamp mode (b)**. Imidacloprid (left graph) induces an ion current for the whole time of application (see bar). After termination of the application the current declines rapidly and returns to the resting level. No depolarization of the cell arises because the membrane potential was clamped by the recording electrodes. In the current clamp mode (right graph) imidacloprid application induces a depolarization of the cell membrane, which triggers the generation of many action potentials.

The agonist effect of imidacloprid on insect nAChR is shown in Figure 2. Application of 0.1 μM imidacloprid opened the nAChRs and induced an inward current under voltage clamp conditions (Figure 2a). This inward current caused a depolarisation in the current clamp mode (Figure 2b). This depolarisation activated the Na^+^-channels, which are the origin of action potentials.

### Demonstration of the synergistic effect of imidacloprid on the flumethrin action *in vitro *at the level of isolated nerve cord fibers

**Figure 3:**
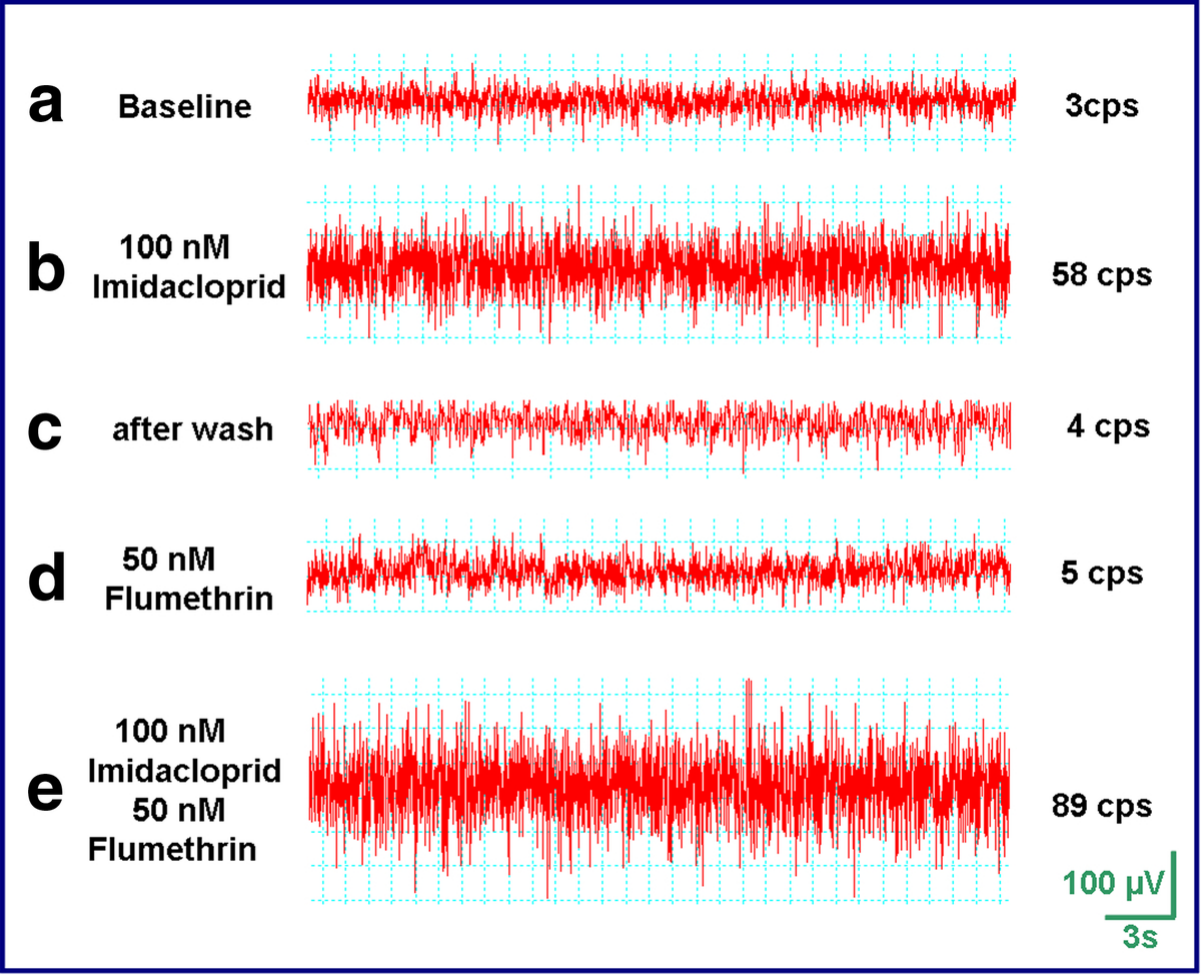
**The synergistic effect of imidacloprid and flumethrin in isolated nerve cord fibres of *Spodoptera frugiperda***. The low baseline spike activity is shown in (a). After the addition of 100 nM of imidacloprid spike activity increased to 58 counts per second (cps). Following washout of the imidacloprid spike activity reduced to 4 cps as shown in (c). The addition of 50 nM of flumethrin alone induced a spike activity of 5 cps (d). The combination of flumethrin and imidacloprid induced a dramatic increase of spike activity to 89 cps (e).

Figure 3 illustrates a typical experiment on the *Spodoptera *nerve cord preparation. The baseline activity for a *Spodoptera *ganglion at rest usually showed a low level of spontaneous spike activity. In the example illustrated in Figure 3a it was 3 counts per second (cps, upper trace) as measured by the differential voltage recordings. After application of a low concentration (100 nM) of imidacloprid, the activity increased within 2-3 minutes, to a higher level of constant activity (58 cps, Figure 3b). This effect was reversible, and the initial baseline activity was again seen within 5-10 minutes after the wash-out of the test compound (Figure 3c). In contrast to imidacloprid, 50 nM flumethrin by itself had no significant effect on the nerve cord activity (5 cps, Figure 3d). Finally, the combination of both compounds induced a higher activity (89 cps) than the sum of the activities induced by either of the compounds alone (Figure 3e).

The results from five independent experiments are summarized in Table 1. Flumethrin (50 nM) alone had negligible effect on the nerve cord activity (5-10 cps) with the exception of one experiment in which the nerve cord activity was increased to 40 cps. Imidacloprid (100 nM) alone increased the nerve cord activity to 50-80 cps. In four of five experiments the combination of both compounds together induced a higher activity (80-100 cps) than the sum of the activities induced by either of the compounds alone.

**Table 1:** The results of 5 independent experiments are summarised

Trial	100 nM Imidacloprid		50 nM Flumethrin		100 nM Imidacloprid + 50 nM Flumethrin		Synergism
	
Trial	Amp[μV]	cps	Amp[μV]	cps	Amp[μV]	cps	
05-05	75	75	-	5	150	100	++

21-05a	50	58	-	5	100	89	++

21-05b	50	75	-	10	70	100	++

25-05a	50	80	10	40	100	90	+/-

25-05b	70	50	-	10	100	80	++

mean	59	67.1	-	14	104	91.8	
	
SD	12.45	12.9	-	14.75	28.81	8.44	
	
P* [vs combination of Flu/Imi]	0.009	0.003	na	0.0007	-	-	

The isolated nerve cord fibres of *Spodoptera frugiperda *were stimulated with 100 nM imidacloprid, 50 nM flumethrin and then a combination of these two compounds and the amplitude of nerve discharge and the number of discharges (counts per second, cps) are given. Imidacloprid alone had a modest effect, flumethrin alone had a negligible effect and the combination of both compounds together demonstrate a clearly synergistic effect well in excess of what either compound alone induced

* Normality and two-sided paired *t*-test was perfomed for all data from five trials using SigmaPlot for Windows 11.0 (Build 11.0.0.75, ^© ^2008 Systat Software, Inc.)

### Coated glass vial contact assay

**Figure 4:**
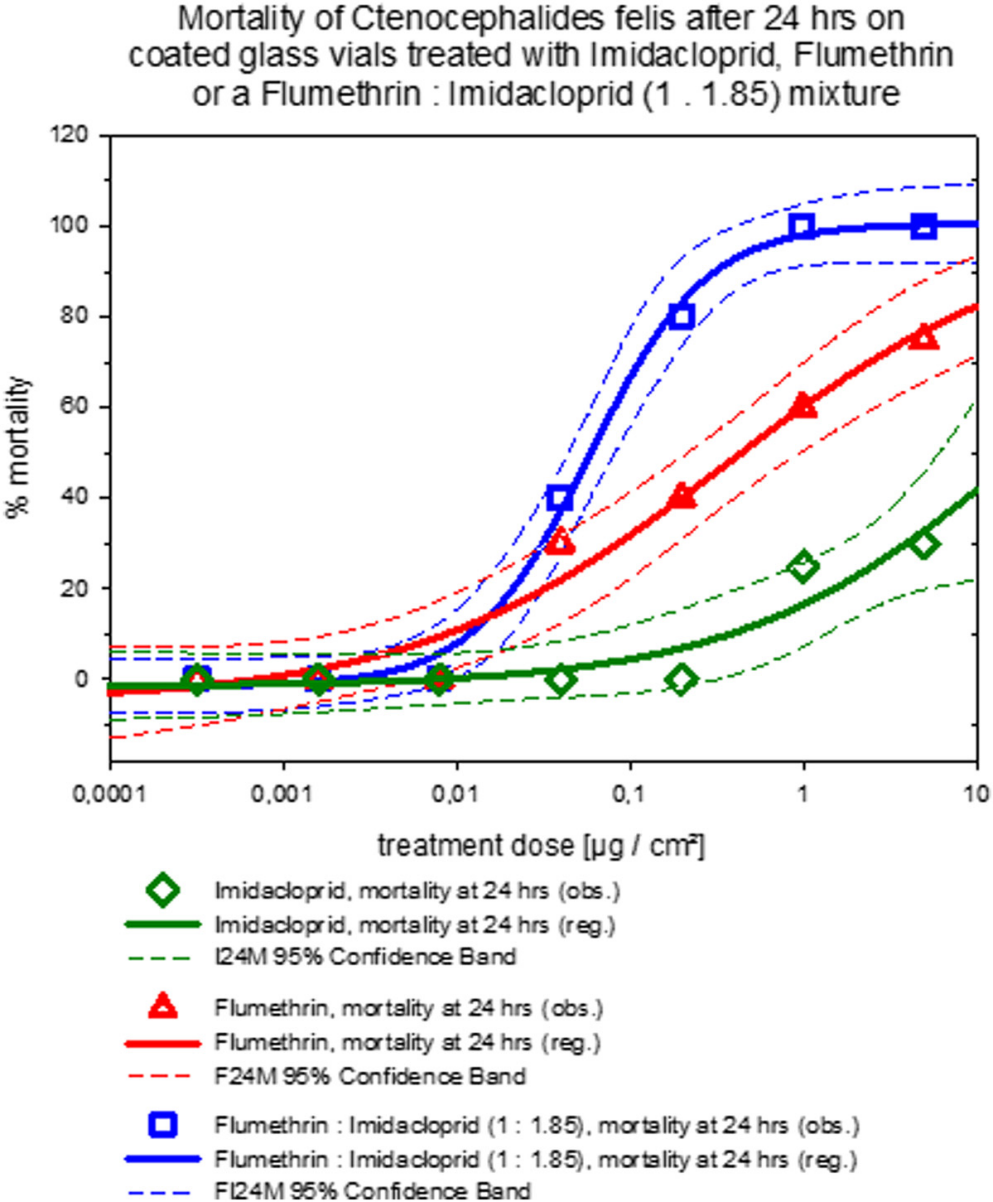
**Dose response curves of flea mortality for flumethrin or imidacloprid or a 1: 1.85 combination of flumethrin and imidacloprid**. The dose response curves show mean % mortality after 24 hours contact against *Ctenocephalides felis *on glass vial surfaces treated with different doses of flumethrin, imidacloprid or a 1: 1.85 combination of flumethrin and imidacloprid (mean data from 2 separate trials; curves including 95% confidence interval as dashed lines calculated with SigmaPlot for Windows 11.0 (Build 11.0.0.75, ^© ^2008 Systat Software, Inc.).

Two trials were conducted and the mean % mortality and efficacy against *Ctenocephalides felis *of flumethrin alone or imidacloprid alone or a 1:1.85 combination of flumethrin and imidacloprid coating on glass vial surfaces were evaluated. Test concentrations of the two compounds were decreased in a step-wise fashion but in the case of the combination, the ratio of the two compounds was kept constant. The combined data and curve fit statistics are provided in Table 2 and the dose response curve for mortality of the combination of the two compounds at 24 hours is illustrated in Figure 4. In summary, it can be said that under the conditions of the coated glass vial contact assay, efficacy against *Ctenocephalides felis *was found to be superior in a 1: 1.85 mixture of flumethrin: imidacloprid over the efficacy of either active alone when considering mortality at 24 hours. Efficacy after 24 hours and efficacy as well as mortality after 48 hours were comparable for flumethrin alone or in combination with imidacloprid. Imidacloprid alone showed slightly poorer performance at 24 hrs in this dry surface *in-vitro *contact assay system.

**Table 2:** The dose response curve showing mean % mortality and efficacy of flumethrin or imidacloprid or a 1: 1.85 combination of flumethrin and imidacloprid coating on glass vial surfaces (mean data from 2 separate trials) against *Ctenocephalides felis *fleas

	Imidacloprid: Flumethrin 1.85: 1				Flumethrin				Imidacloprid			
**[μg/cm**^**2**^**]**	**24 h**		**48 h**		**24 h**		**48 h**		**24 h**		**48 h**	

	**Mortality**	**Efficacy**	**Mortality**	**Efficacy**	**Mortality**	**Efficacy**	**Mortality**	**Efficacy**	**Mortality**	**Efficacy**	**Mortality**	**Efficacy**

5	100	100	100	100	75	100	100	100	30	100	100	100

1	100	100	100	100	60	100	80	100	25	90	100	100

0,2	80	100	100	100	40	100	70	100	0	60	80	100

0,04	40	80	60	100	30	60	60	100	0	30	40	100

0,008	0	30	30	60	0	0	0	40	0	0	0	0

0,0016	0	0	0	0	0	0	0	0	0	0	0	0

0,00032	0	0	0	0	0	0	0	0	0	0	0	0

0,000064	0	0	0	0	0	0	0	0	0	0	0	0

min	-1,582	-2,287	-0,078	-0,011	-4,929	-1,243	-3,390	0,031	-1,531	-0,914	-1,752	-0,012

max	100,5	100,4	103,8	100,0	100,0	99,9	93,7	100,0	100,0	101,0	106,0	100,0

ED50 μg/cm^2^	0,059	0,012	0,033	0,008	0,483	0,010	0,039	0,009	0,537	0,074	0,112	0,017

Slope	1,296	1,285	1,111	3,889	0,363	1,736	1,031	4,006	17,378	1,433	0,937	3,315

ED50 Factor (FI = 1)					8,2	0,9	1,2	1,2	9,1	6,4	3,4	2,3

Normality* Test (Shapiro-Wilk)	Passed	Passed	Passed	Passed	Passed	Passed	Passed	Passed	Passed	Failed	Passed	Passed

*P*-values* 24 h mortality vs untreated control	0.028				0.004				0.0457			
											
*P*-values* FI vs F or I	-				0.0094				-0.0024			
											
*P*-values* F vs I	-				-				-0.0027			

* Normality and two-sided paired *t*-test was perfomed for data used in Figure 4 with all data from both trials, means of the two trials were used for curve fitting and fit statistics using SigmaPlot for Windows 11.0 (Build 11.0.0.75, ^© ^2008 Systat Software, Inc.)

At 24 hrs imidacloprid induced flea mortality in this dry surface contact test was just significantly different from the control group (*p *= 0.0457), the flumethrin group (*p *= 0.004) and the combination treatment with 1:1.85 flumethrin: imidacloprid (*p *= 0.0028). The combination treatment was also significantly different from the imidacloprid (*p *= 0.0024) and flumethrin (*p *= 0.0094) single treatments. The flumethrin treatment was also significantly different from the Imidacloprid treatment (*p *= 0.0027) thus supporting the synergistic effect found in the electrophysiological measurements on isolated insect neurons.

**Figure 5:**
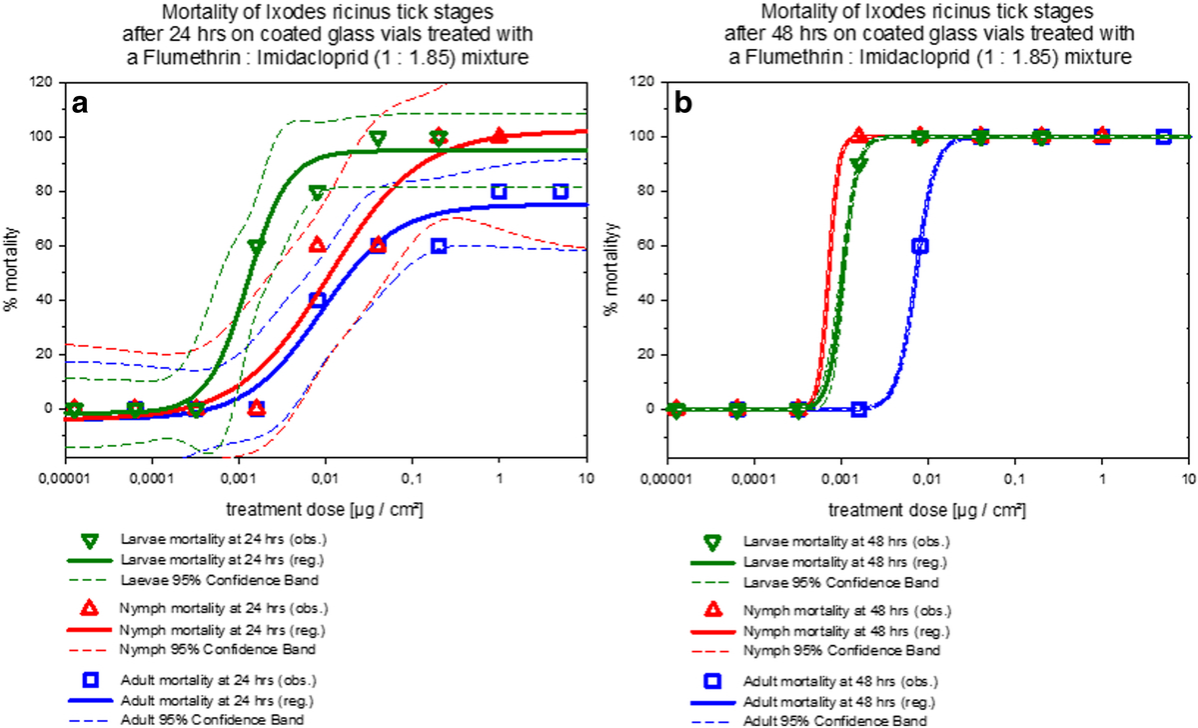
**Dose response curves for flumethrin: imidacloprid (1: 1.85) mortality against *Ixodes ricinus *life stages in coated glass vial contact assays**. **a) **Dose response curves after 24 hours contact time. **b) **Dose response curves after 48 hours contact time. *Note: *The curves include the 95% confidence interval as dashed lines calculated with SigmaPlot for Windows 11.0 (Build 11.0.0.75, ^© ^2008 Systat Software, Inc.).

**Figure 6:**
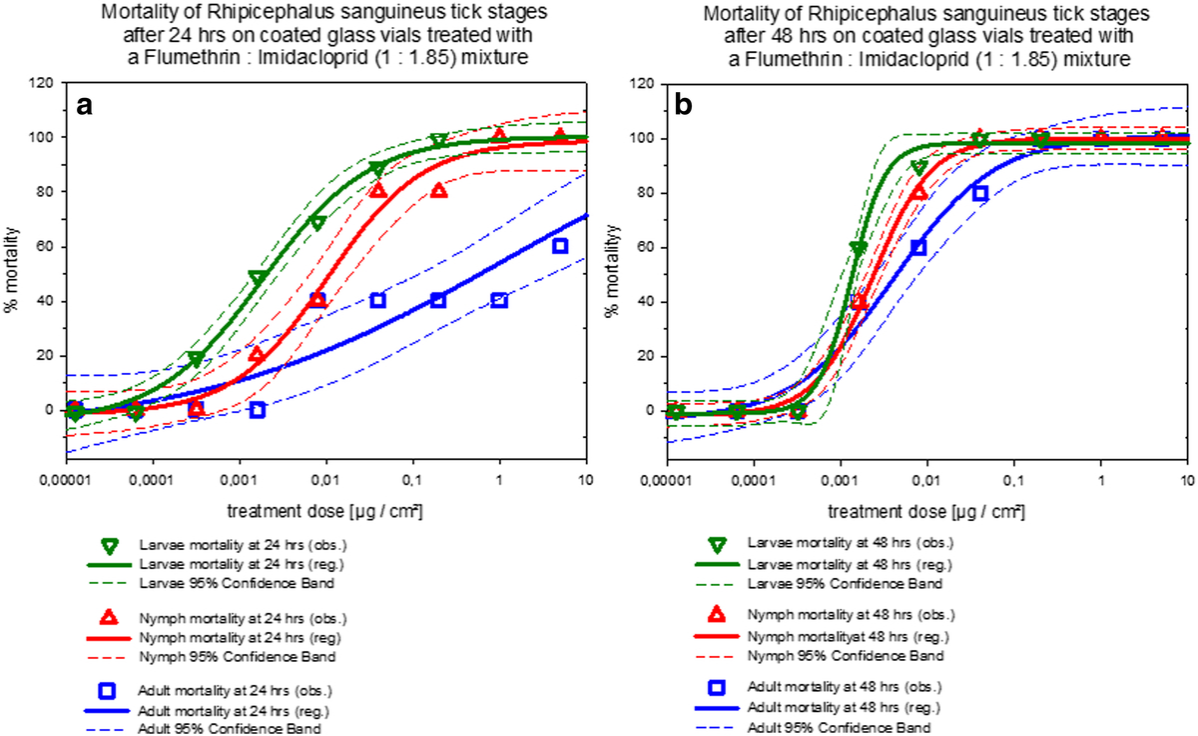
**Dose response curves for flumethrin: imidacloprid (1: 1.85) mortality against *Rhipicephalus sanguineus *life stages in coated glass vial contact assays**. **a) **Dose response curves after 24 hours contact time. **b) **Dose response curves after 48 hours contact time. *Note: *The curves include the 95% confidence interval as dashed lines calculated with SigmaPlot for Windows 11.0 (Build 11.0.0.75, ^© ^2008 Systat Software, Inc.).

**Figure 7:**
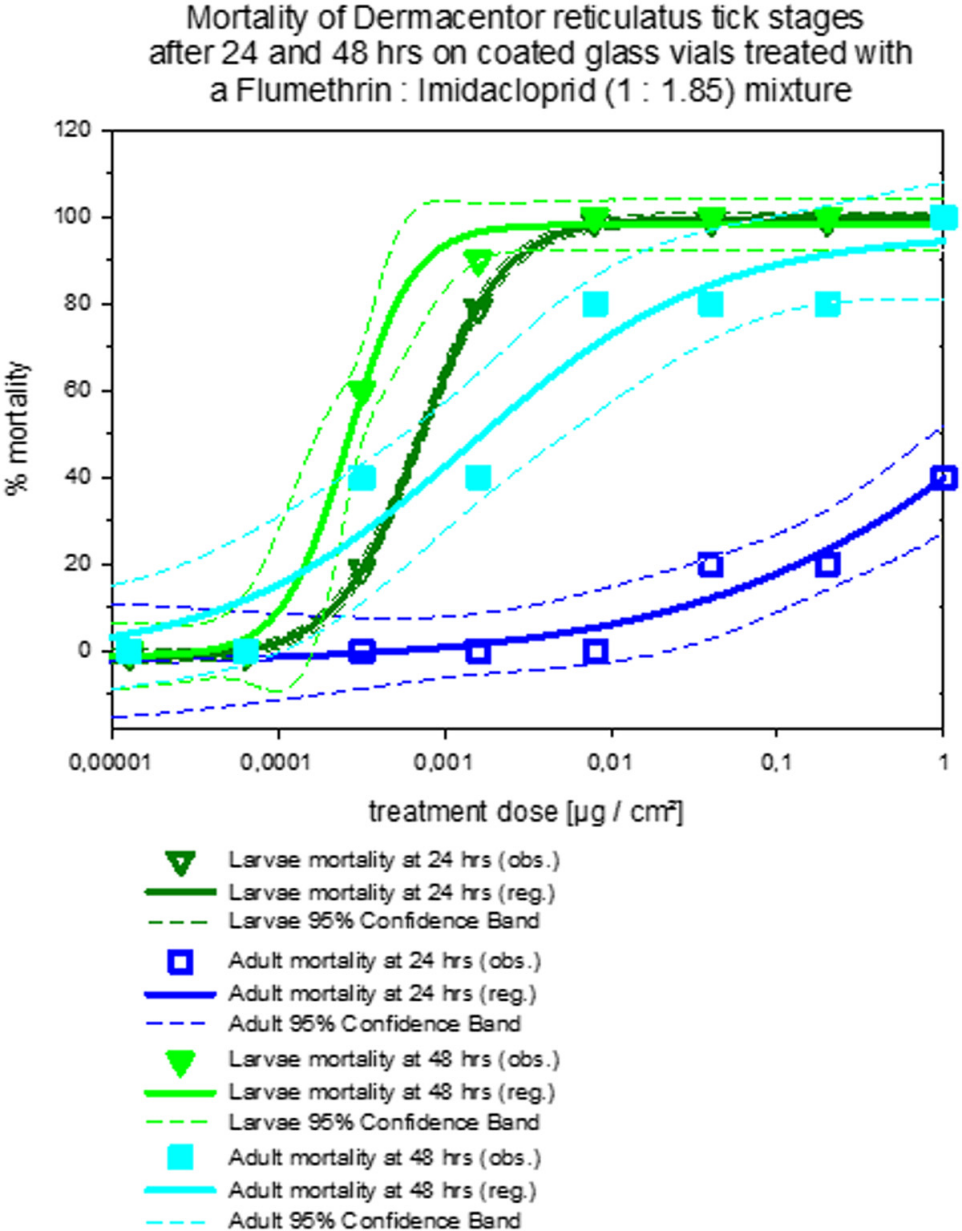
**Dose response curves for flumethrin: imidacloprid (1: 1.85) mortality against *Dermacentor reticulatus *stages in coated glass vial contact assays**. Mortality dose response curves after 24 and 48 hours contact time. *Note: *The curves include the 95% confidence interval as dashed lines calculated with SigmaPlot for Windows 11.0 (Build 11.0.0.75, ^© ^2008 Systat Software, Inc.).

Figures 5, 6 and 7 show that the effect of stage dependent susceptibility to the 1: 1.85 flumethrin: imidacloprid mixtures is most pronounced for the mortality curves after 24 hrs for all tick species tested. With *Ixodes ricinus *the slopes of the curves were shallower and adults show similar susceptibility as nymphs. For *Dermacentor reticulatus *only larvae and adults were tested. While larval susceptibility and adult mortality did not change significantly between 24 and 48 hours incubation time, adult mortality did increase.

It may thus be concluded that the 1: 1.85 flumethrin: imidacloprid mixture produced mortality curves that varied dependent on the tick life stage and tick species tested although significant mortality was achieved throughout at biologically relevant compound concentrations. Comparing tick species mortality curves of the same developmental stage after treatment with of the 1: 1.85 flumethrin: imidacloprid mixture it may be concluded that in general *Dermacentor reticulatus *larvae and adults were more susceptible to the 1: 1.85 flumethrin: imidacloprid mixture than *Ixodes ricinus *(all life stages) followed by *Rhipicephalus sanguineus *(all life stages) under the conditions of this trial.

All tick treatment groups were significantly different from the controls (control mortality zero throughout the trial in all species) regardless of tick species or developmental stage (*p *< 0.001). Considering the mortality data of *Rhipicephalus sanguineus*, larvae were more susceptible than adults (*p *= 0.006) and nymphs (*p *= 0.021), and nymphs were more susceptible than adults (*p *= 0.021). The *Ixodes *mortality curves of nymphs and larvae were not significantly different from each other (*p *= 0.201). *Ixodes ricinus *adults were less susceptible than nymphs (*p *= 0,034) and larvae (*p *= 0.034). Most pronounced was the difference between adult and larval *Dermcentor reticulatus *mortality (*p *< 0,001).

### Weight loss of collars over time

**Figure 8:**
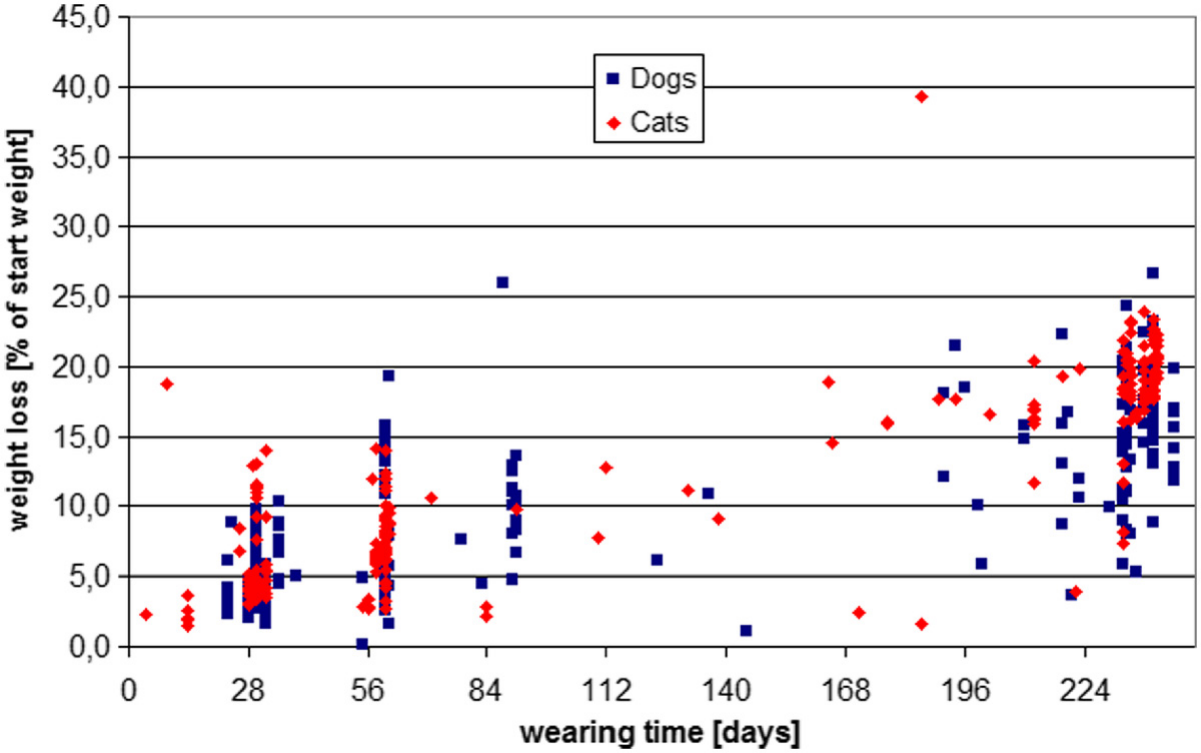
**Weight loss of collars in cat and dog studies as calculated at various time points over the duration of the study**. The weight loss is presented as % of the starting weight. 223 cat collars and 236 dog collars were included. Single unusually high values reflect chewed collars.

The percentage weight loss of the collars over time in the cat studies are reflected in Table 3. The percentage of weight loss of the collars over time in the dog studies are reflected in Table 4. Summary data is provided in a dot plot in Figure 8.

**Table 3:** The percentage weight loss of collars in the cat studies over time

Bayer Animal Health study member	Breed (body weight [kg])	Study duration [days]	mean weight loss [%]
146.164	Mix (2.3-5.4)	30	10,7
		
		238	20,1

146.303	DSH (3.7-4.7)	238	18.5

146.159	Mix (2.1-4.7)	60	8,4

152.152	DSH (2.8-5.3)	61	9.1

152.150	DSH (0.86-1.47) SD 0	30	5,04
		
		60	7,10

146.747	DSH (3.6-5.7)	28	4.4

146.156	DSH (2.8-5.3)	238	19.1

146.045	DSH (3.8-7.0)	241	20.8

146.155	DSH (2.7-	240	18.8

146.162 (field)	several (1.7-8.3)	up to 240 days	time depending

Small collars were used in a total of 157 cats in laboratory based studies plus 66 cats in a field study. DSH: domestic short haired

**Table 4:** The percentage weight loss of collars in the dog studies over time

Bayer Animal Health study number	Breed (body weight [kg])	Study duration [days]	mean weight loss [%]
146.165	Mix (11.6-19.4)	30	18.1
		
		238	5.4

146.306	Beagle (9.4-13.6)	238	17.8

146.161	Mix (8.4-20.2)	60	4.2

152.151	Beagle (8.7-13.4)	61	7.9

152.149	Beagle (1.5-2.9) SD 0	23	3,4
		
		30	6.5
		
		60	13.5

146.737	Beagle (8.7-11.7)	28	3.2 and 2.9

146.158	Beagle (8.8-12.8)	245	14.8

146.390	Beagle (6.6-20-4)	240	16.0

146.592	Beagle (8.3-13.5)	240	17.7

146.269	Mix (7.1-21.5)	90	8.3

146.607	Beagle (10.0-13.1)	91	9.7

146.163 (field)	collar S (n = 48; of 45 dogs): several breeds (0.5-8 kg)	up to 240 days	time depending
			
	collar L (n = 242; of 219 dogs): several breeds (8-69 kg)		time depending

Large collars were used in all the laboratory based animals totalling 177 animals plus 59 field study animals. Of these, 48 used large collars and the remainder used small collars.

### Surface content on the collar over time

**Figure 9:**
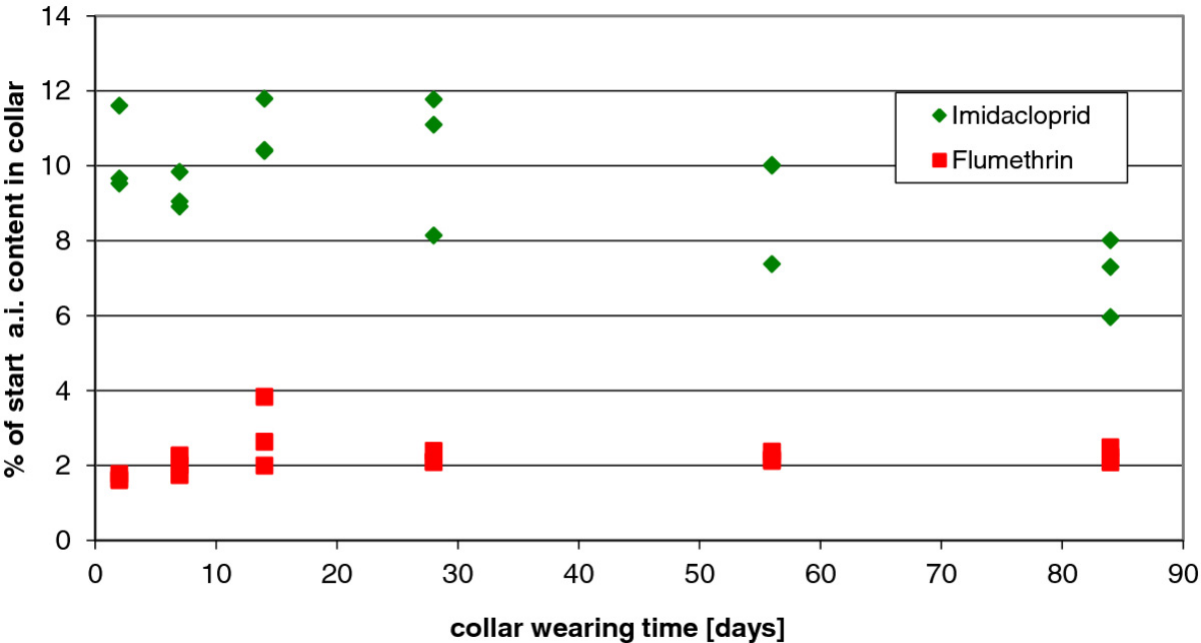
**Surface content of imidacloprid and flumethrin on the surface of collars after being worn for various periods of time**. The figure reflects the removable surface content on the collar surfaces worn by cats for 2, 7, 14, 28, 56, and 84 days. 3 collars were tested per time point.

Results of the surface analysis immediately after collar removal at various time points after collar fitting show that both active ingredients were present on the surface of the collars worn by cats over the whole investigation period. By day 2 the imidacloprid surface content was at approximately 9-12% of the starting collar content, and decreased slightly to 6-8% of the starting content by day 84. There was a relatively broad scattering of the surface content of imidacloprid over the time period of study. The content of flumethrin on the surface of the worn collars was, however, relatively constant during the study with a slight increase by day 14 which then plateaued at around 2% until day 84 (Figure 9).

### Release of active ingredients over time

**Figure 10:**
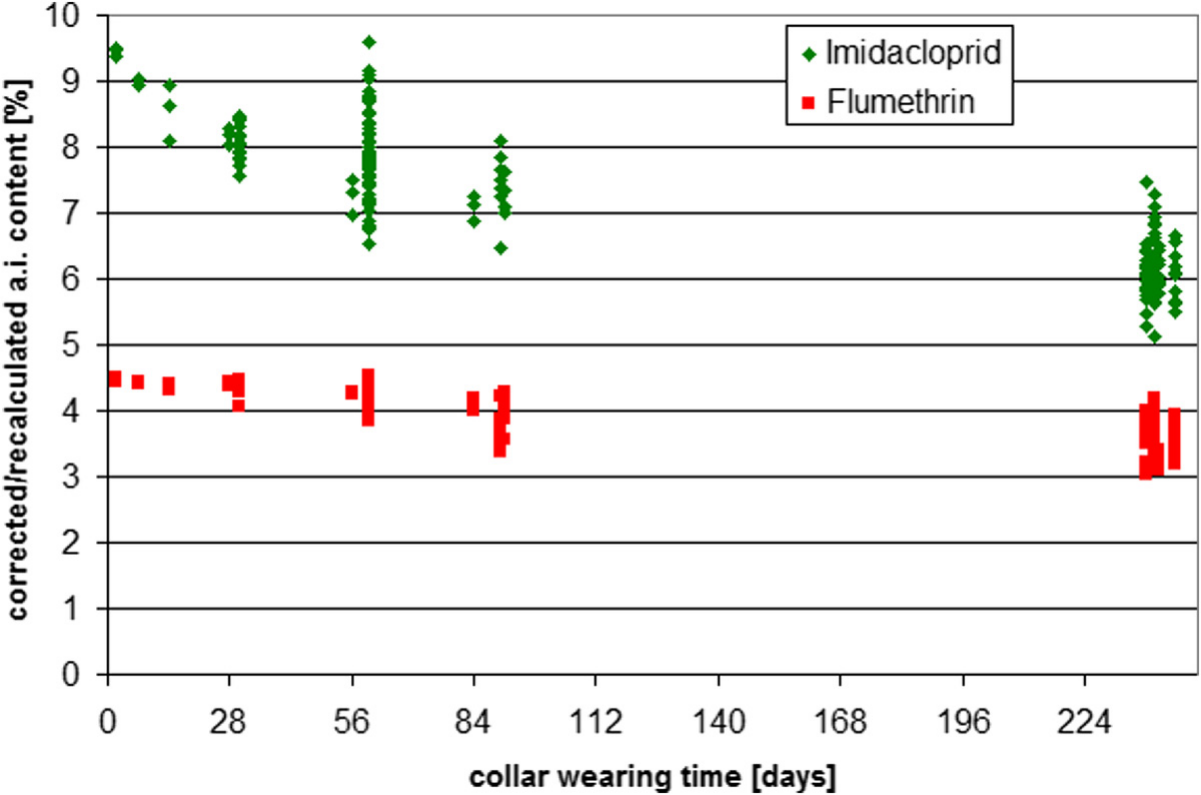
**Decrease of imidacloprid and flumethrin collar content over time**. The figure describes the decrease of the relative imidacloprid and flumethrin collar content in 177 collars worn by 93 cats and 84 dogs for different durations during laboratory studies.

Determining the remaining content of active ingredients in the collar over time and the amount of active ingredients released from the collar provided the best indication of the kinetics of release of the compounds from the collar matrix over the duration of the study. These data were calculated as relative remaining amount [%] compared to the initial starting amount of both active ingredients and given as mean values per study (Table 5). They were further re-calculated as relative collar content and are shown in a dot plot in Figure 10 for both active ingredients.

**Table 5:** The remaining percentage (± standard deviation) of the imidacloprid and flumethrin start amount in 177 collars worn by laboratory cats and dogs for different durations

Bayer Animal Health Study number	No. of animals	Wearing time [days]	mean % of initial amount	
			
			Imidacloprid	Flumethrin
CAT STUDIES = small collar				

ID 35118	3	2	94.6 ± 0.67	99.7 ± 0.79
	
	3	7	89.6 ± 0.48	98.3 ± 0.44
	
	3	14	85.5 ± 4.32	97.0 ± 1.25
	
	3	28	81.7 ± 1.32	97.7 ± 0.75
	
	3	56	72.6 ± 2.61	95.0 ± 0.22
	
	3	84	70.9 ± 1.81	91.7 ± 2.27

ID 35638	8	30	79.7 ± 2.95	97.0 ± 0.64

ID 33697	30	60	77.7 ± 8.30	92.5 ± 3.13

ID 35121	8	238	74.7 ± 4.53	81.6 ± 5.39

ID 35635	10	241	61.4 ± 2.50	72.7 ± 2.52

ID 35632	10	240	57.9 ± 3.27	85.6 ± 2.28

ID 35630	9	238	59.7 ± 3.12	84.9 ± 2.23

***mean cat 8 months ***	***37 ***	***238-240 ***	***59.7 ± 3.0 ***	***81.9 ± 6.0 ***

DOG STUDIES = large collar				

ID 35637	8	30	81.7 ± 2.23	96.5 ± 2.97

ID 33692	32	60	79.1 ± 4.62	97.7 ± 2.71

ID 33691	10	90	74.7 ± 4.53	81.6 ± 5.39

ID 35117	6	91	72.0 ± 2.32	88.8 ± 5.92

ID 35631	10	245	60.5 ± 3.96	80.8 ± 5.44

ID 35120	8	238	62.4 ± 6.21	70.4 ± 3.47

ID 35636	10	240	68.7 ± 2.03	80.8 ± 7.40

***Mean dog 8 months ***	***28 ***	***238-245 ***	***64.0 ± 5.40 ***	***77.8 ± 7.2 ***

**Mean total 8 months**	**65**	**238-245**	**61.6 ± 4.70**	**80.1 ± 6.9**

The results show a slow and steady release of imidacloprid and flumethrin to beyond 8 months at which time the collars contained approximately 60% (imidacloprid) and 80% (flumethrin) of the start concentration. The collar applications were well tolerated in all cats and dogs and no clinical signs of disease or adverse effects besides mild mechanical effects were noted when collars were applied correctly.

### Acaricidal efficacy of cat and dog hair over time

All control ticks on 100% of the untreated hair samples were alive, healthy and showed normal behaviour for the duration of the incubation period. In contrast to this, all hair samples collected from cats and dogs treated with the collar over the period of 8 months caused pronounced tick mortality. After 4 hrs of contact time ticks appeared generally disorientated and showed ataxic movements but no acaricidal efficacy was observed. After 6 hrs of contact time 97.9% of the ticks were dead or moribund, 0.5% of the ticks showed ataxic or uncoordinated movement whilst only 1.7% of the ticks were alive and appeared normal. This was also true after 8 hrs of contact time. After 12 hrs of contact time 99.8% ticks were dead or moribund, 0.2% of the ticks were ataxic. Finally, after 24 hrs of contact time all ticks were dead or moribund, making the 48 hr evaluation meaningless.

The results of a single experiment representing the 6 hr (which were unchanged at 8 hrs) and 12 h (which were unchanged at 48 hrs) evaluation are presented in Table 6.

**Table 6:** The 6 and 12 hour incubation efficacy of hair coat samples of dogs and cats wearing imidacloprid 10%/flumethrin 4.5% collars for 8 months, on adult *Rhipicephalus sanguineus *ticks

Sampling day = Day after treatment			7	14	21	30	59	90	120	149	181	210	240
killing^1^ efficacy	cat [%]	6 hours	100	83	100	100	100	100	100	67	33	0	50
			
	dog[%]		100	67	100	83	100	100	100	100	100	100	83
		
overall^2^ efficacy	cat [%]		100	83	100	100	100	100	100	67	33	100	50
			
	dog[%]		100	100	100	100	100	100	100	100	100	100	83

killing^1^ efficacy	cat [%]	12 hours	100	100	100	100	100	100	100	100	83	100	100
			
	dog[%]		100	100	100	100	100	100	100	100	100	100	100
		
overall^2 ^efficacy	cat [%]		100	100	100	100	100	100	100	100	100	100	100
			
	dog[%]		100	100	100	100	100	100	100	100	100	100	100

^1 ^killing efficacy was calculated from ticks being dead or moribund^2 ^overall efficacy was calculated from ticks being dead or moribund + atactic ticks

## Discussion

Pharmacologic synergism is not a new concept and may be defined as the combined effect of two (or more) substances that have a significantly greater effect than the effects of the individual substances used independently [34]. Pharmacological synergism was in fact first described in ectoparasiticides [35,36] but is also widely appreciated in antimicrobial therapy [37] and in cancer chemotherapy [38]. Flumethrin and imidacloprid have never previously been used together in a single preparation for ectoparasite control in dogs and cats. Both compounds have a neuropharmacological excitatory effect but through differing molecular mechanisms and it may be speculated, based on their pharmacological mechanism of action that they would act synergistically against fleas and ticks. Neural cell membrane sodium channels are present in three different states: closed, open, and inactivated (Figure 1a). Flumethrin preferentially binds to the open state channel and then keeps the channel open resulting in a state of persistent depolarization. A low dose of imidacloprid would activate nicotinic acetylcholine receptors (nAChR) channels inducing an inward flow of current causing depolarization of the cell and activating (thus opening) sodium channels thus theoretically making them significantly more susceptible to the excitatory (and hence toxic) effects of flumethrin.

The initial investigation made use of an *in vitro *isolated insect nerve study. At rest, the isolated *Spodoptera frugiperda *ganglia usually show a low level of spontaneous spike activity of between 3 and 25 counts per second (cps). This rate increases dramatically when compounds that are known to increase the excitability of the neurons by activating of nAChR, such as imidacloprid, are applied. In contrast to this, flumethrin does not increase the activity of an almost silent nerve bundle, which could be plausibly explained by the fact that pyrethroids bind preferentially to open sodium channels (i.e. an activated neuron) and therefore will have no or little effect on a resting neuron (at least at the low concentrations used in this study). It is known that in insects the pyrethroid insecticides (such as flumethrin) bind preferentially to the open (activated) sodium channel and once bound keep the channel open [39]. The action of pyrethroids is thus described as 'use dependant' in that they have little effect on resting nerve cells but a marked effect on stimulated cells. A low dose of imidacloprid activates nAChR channels and increases the frequency of the nerve cell electrical spiking. This translates into more sodium channels being open thus allowing flumethrin far better interaction with its target. Thus, as we have shown here, the application of both compounds together leads to a much higher level of activity than either compound alone, which demonstrates that neonicotinoid insecticides (such as imidacloprid) acting on the nAChr and pyrethroid insecticides acting on voltage dependent sodium channels cooperate at the level of the insect nervous system to produce an enhanced sensitivity to the pyrethroid component, providing a way to obtain similar levels of pest control with a reduced dose of pyrethroid. This is a classical (albeit new) example pharmacological synergism.

The next phase of the study investigated whether this principle would be visible in a well-controlled *in vitro *tick and flea study. The glass vial (dry surface) efficacy studies were conducted as an *in vitro *measure of flea and tick sensitivity to the flumethrin and imidacloprid and their combination. Using an *in vitro *system prior to assessment in an *in vivo *system provides accurate and repeatable data on the effect of the active compounds themselves without the added variable of many uncontrolled factors that a dog or cat skin and hair coat would add. What the *in vitro *isolated *Spodoptera frugiperda *ganglia studies showed in respect of the synergistic effects of imidacloprid and flumethrin was observed in this glass vial study on the whole living tick and flea organism. Although the *in vitro Spodoptera *nerve study demonstrated the synergism was true in an insect system, the efficacy studies conducted in the glass vial system indicated that the combination product exerted very high activities against ticks as well with *Dermacentor *and *Ixodes *being the most sensitive species followed by *Rhipicephalus sanguineus*.

Having demonstrated that the theory of pharmacological synergism holds true in an isolated insect nerve study and against the ticks and fleas in a glass vial system, the next phase of the study reported here evaluated the behaviour of the active ingredients in a collar matrix application device. The advantage of a collar system is the ease of application for an owner (enhancing compliance) and the potential for continuous slow release of the actives onto the dog or cats coat over an extended period of time (encouraging owner compliance further). The first way of evaluating this was through simply monitoring weight loss of the collar over time. The collars lost between 15 and 18% of their weight on the dogs and between 19 and 20% of their weight on the cats when worn over the full period for which insecticidal and acaricidal amounts of the actives are released (8 months). The fastidious grooming behaviour of cats could explain the slightly increased weight loss of the collar on the cat compared to the dog. Grooming behaviour would remove actives from the coat and result in a higher concentration gradient and hence faster collar reload of the coat in the cat than the dog.

The next way to evaluate the release kinetics of the actives from the collar was to evaluate the concentration of the actives on the surface of the collar at various time points. The results of the surface concentration of the active ingredients over time demonstrated an effective surface reload onto the collar surface that allowed for a reload of the actives down a concentration gradient from the collar surface to the animal's coat. Results of the surface analysis immediately after collar removal at various time points after collar fitting showed that approximately 2% of the starting flumethrin content in the collar is present on the collar surface for the 8 month study duration. The actives on the surface of the collar are constantly unloading onto the animal's hair coat and a dynamic equilibrium is established between actives in the collar, on the collar surface and on the animal's coat. The imidacloprid on the surface of the collars decreased slightly over the 84 days of the study from 9-12% of the starting collar content, to 6 - 8% at day 84, which suggests that removal of the active ingredient down the concentration gradient from the collar to the surface to the animal's hair coat is slightly faster than what the collar releases as actives onto its surface. These results are supported by the analysis of the concentrations of the actives in the collar over time. The change in concentration of the active ingredients in the collar matrix over the duration of the collar application in dogs and cats indicated a steady decrease due to a release of the active compounds for a time period slightly in excess of eight months. By the termination of the experiments (at 8 months) collars contained approximately 60% of their starting imidacloprid content and 80% of their flumethrin starting content. This reflects a continuous concentration gradient between the collar and the coat and a continuous coat reload of the actives from the collar for the 8 months of collar application. To substantiate a claim of efficacy of 8 months, the amount of active ingredients remaining in the collar at 8 months should still result in a sufficient concentration gradient between collar and animal surface to allow for steady release and biological efficacy for another several months beyond the claim. When evaluating efficacy against ticks and fleas in the real life situation in the numerous studies reported by Stanneck [25,26] there was a decrease in efficacy, however, to below a 90% (for ticks) or 95% (for fleas) after 8 months. This is reflected in the product's registered label claim. This apparent contradiction (a drop in efficacy to below threshold) despite adequate collar concentrations of the active compounds) reflects the delicate balance of the physical and -chemical environments at play in such a long-term collar release matrix. These factors are influenced by numerous variables and as such are much more complex than the factors in play in the application of an active in a single-application fluid system such as with a spot on.

Finally, following evaluation of *in vitro *efficacy of the combination of imidacloprid and flumethrin and evaluation of the behaviour of these actives in the collar, an experiment evaluated the efficacy of hair clipped from dogs and cats wearing the collar against ticks and fleas. It has been known for many years now that the concentration of imidacloprid achieved on the coat of spot on treated animals is effective up until the end of the 4^th ^week after application (the duration for which efficacy is claimed). Keeping the levels in the coat achieved by the spot on product in mind, we assumed that because the levels achieved by the collar device described here were never below those achieved by the spot on (for 8 months), the collar delivered imidacloprid dose would remain effective for this period. Topical flumethrin has long been known to be an effective acaricide, but the hair coat concentrations for efficacy are unknown. It has previously been established that it does not work systemically as topically applied compound is below the limit of detection in the blood of topically treated animals (Bayer Animal Health registration study ID 35642 and ID 35643, unpublished data). Taken together these unpublished results necessitated an *in vitro *study that evaluated the efficacy of hair harvested from dogs and cats treated with the imidacloprid: flumethrin collar device. Special attention was given to efficacy at very early time points (starting at 4 hours post collar application). Preventing tick attachment is crucial to preventing disease transmission. Including ticks that show abnormal behaviour that would prevent them from normal attachment and feeding, hair harvested from collar treated dogs and cats showed an efficacy of 99.3% after 6 hours and 100% after 12 hours (and hence for any time point thereafter for the duration of 8 months). These results strongly support a non-systemic, obviously external mode of action over the period of 8 months and provide evidence of very rapid repellent acaricidal efficacy which has also been reported from numerous other on animal studies [25-27,40]. This is the longest time period for which a collar impregnated with an acaricide and an insecticide together has ever been shown to be capable of providing therapeutically relevant concentrations of active ingredients from its surface to the coat of a dog or cat.

## Conclusions

In conclusion, we have been able to demonstrate a unique pharmacological synergism between 10% imidacloprid and 4.5% flumethrin. This synergism was shown in an *in vitro *isolated insect nerve study and in an *in vitro *glass vial study using various tick life stages in various tick species and in fleas. The release kinetics of the active ingredients from the collar were also evaluated and it was found that therapeutically relevant doses of both imidacloprid and flumethrin were released from the collar for 8 months. Hair harvested from collar treated dogs and cats showed a very early (6 hours) repellent activity that would prevent ticks from attaching and feeding, thus minimizing the opportunity a tick would have to attach, feed and transmit disease. This is the longest a collar device has ever shown this release kinetic. The 8 month long duration of efficacy after a very simple collar application greatly simplifies the pet owner's task of ectoparasite control in a world where owner compliance is resoundingly poor. This is largely due to limited veterinary instruction and the relatively intensive managemental responsibility placed on owners for frequent product applications all year around in environments where vector borne diseases are not declining but are rather an increasing threat to both animal and human health.

## Competing interests

These studies were completely funded by Bayer Animal Health GmbH, Monheim, Germany, of which D. Stanneck, E. Kruedewagen, A. Turberg, W. Jiritschka and K. Krieger are employees. The only exception is the neurophysiological work which was funded and conducted by Bayer CropScience AG of which U. Ebbinghaus-Kintscher and E. Schoenhense are employees. A. Leisewitz is Professor of Companion Animal Clinical Studies at the Faculty of Veterinary Science, University of Pretoria, South Africa. All authors voluntarily publish this article and have no personal interest in these studies other than publishing the scientific findings that they have been involved in planning, conducting, monitoring and analysing.

## Authors' contributions

DS and EMK designed the study for all experiments involving on animal evaluations and compiled and analysed the data deriving from these studies. UEK and ES were responsible for the neurophysiological part of the study. AT conducted the arthopod *in vitro *experiments. WJ was responsible for the analytical work on the collars. AL was responsible for the drafting of the manuscript which was substantially edited by all authors. All authors read and approved the final manuscript.
